# Inflammatory Myofibroblastic Tumor of the urinary bladder: A case report

**DOI:** 10.1016/j.eucr.2024.102726

**Published:** 2024-04-24

**Authors:** Samuel Lassiter, Michael L. Smith, James Siegert, Thai Nguyen

**Affiliations:** a Lake Erie College of Osteopathic Medicine, United States; b Franciscan Health Olympia Fields, United States; c Advanced Urology Associates, Joliet, IL

## Abstract

Inflammatory Myofibroblastic Tumors represent a rare subset of spindle cell tumors that can occur in the genitourinary tract, most commonly within the bladder. These tumors are proposed to fall in a spectrum of benign inflammatory pseudotumors to true sarcomas. This, along with their rarity makes diagnosis and treatment challenging to clinicians. We present a 51-year-old female diagnosed with IMT of the bladder following a presentation for hematuria. Treatment consisted of transurethral resection of the tumor. This case, and the accompanying review of the literature highlight the need for further research due to lack of clarity for diagnosis and treatment.

## 1 Introduction

Inflammatory Myofibroblastic Tumors (IMT's) are a rare subset of spindle cell tumors that can occur in the genitourinary tract, most commonly within the bladder.^1^ IMT's of the bladder provide a challenge to clinicians for both diagnosis and treatment with a spectrum of disease ranging from more benign pseudosarcomatous proliferations to more aggressive lesions.^2^ These tumors have been most reported in patients aged 35–55, with the most common presenting symptom being hematuria, followed by urinary frequency, dysuria, and obstructive symptoms.^3^ In this case, we present an IMT discovered in a 51-year-old female.

## 2 Case presentation

**Fig. 1:**
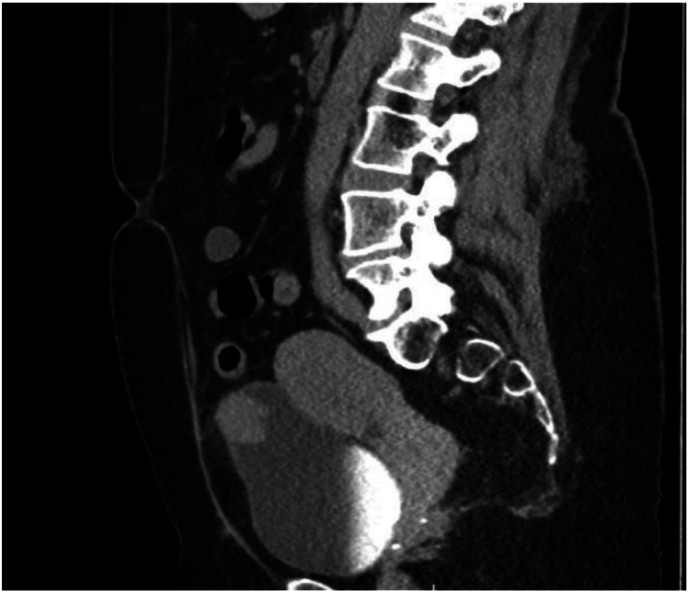
CT Urogram.

**Fig. 2:**
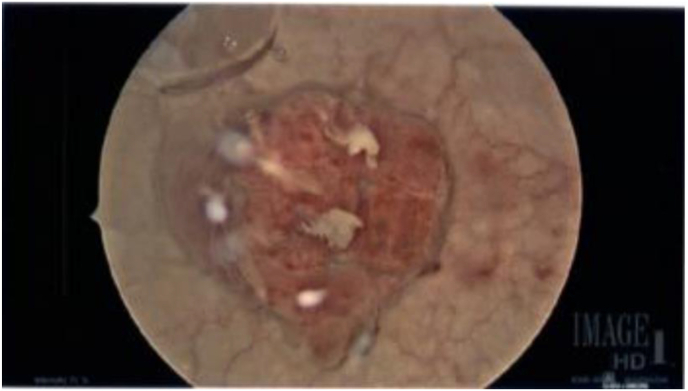
Cystoscopic image immediately prior to resection.

A 51-year-old female with a past medical history of anxiety, depression, and past surgical history of cholecystectomy, cesarean section, and salpingo-oophorectomy with appendectomy, presented to the emergency department with a chief complaint of gross hematuria. Shortly prior to her presentation a CT of the abdomen and pelvis without contrast was performed at an outside facility which demonstrated a 2.7 cm lesion at the left anterior dome of the bladder. Following admission, she underwent a CT Urogram (Fig. 1) redemonstrating the bladder mass without any other urological findings that could explain the hematuria. During hospitalization, the hematuria improved and she was discharged with plans for outpatient intervention. She subsequently underwent transurethral resection of bladder tumor in the ambulatory setting. Cystoscopic image of the lesion before resection can be seen in Fig. 2. Specimen from the tumor demonstrated the abscence of invasion and positive immunohistochemical stains for ALK-1, but negative for P40, CK34, CK5/6, Desmin, and PAX8 upon pathology evaluation. Ultimately, the tumor was determined to be an inflammatory myofibroblastic tumor.

**Fig. 3:**
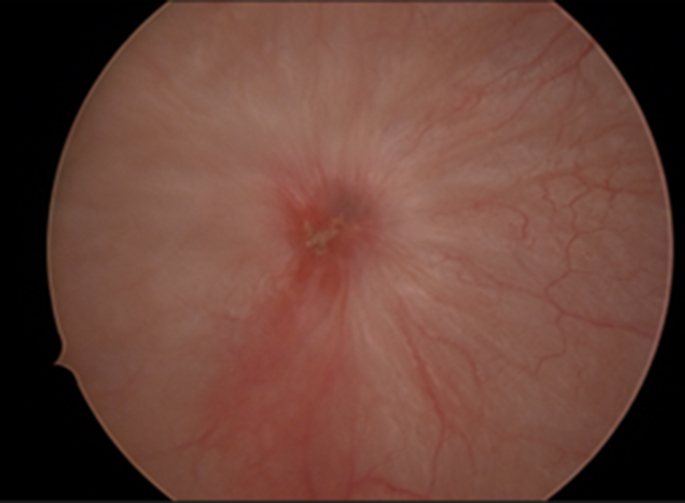
Cystoscopic image 9 months following initial TURBT.

A variety of treatment options were discussed with the patient including surveillance cystoscopy, repeat TURBT, and partial cystectomy, given the uncertain behavior of lesion. In this case, the patient elected to proceed with continued surveillance via cystoscopy. Follow-up cystoscopy (Fig. 3) with cold - cup biopsy nine months after initial surgery reveal partially denuded urothelial mucosa on pathological analysis, however no residual tumor was identified. Cystoscopy three months following this revealed no further tumor, and complete healing of the urothelium.

## 3 Discussion

Inflammatory Myofibroblastic Tumors were first discovered by Brunn in 1931 and described as a pulmonary myoma.^4^ Since that time, IMT's have been described in the lungs, pancreas, prostate, testicles, and bladder.^5^ The first report of an IMT in the genitourinary system occurred in 1980, with the bladder now recognized as the most common location within the GU tract.^6^ More generally, IMT's were initially classified as inflammatory pseudotumors, however further analysis has raised suspicion that they have a neoplastic basis.^7^ These tumors present a unique problem to practicing urologists, as their rarity has yielded limited data for appropriate diagnostic criterion, as well as the most effective treatment modality. While generally regarded as a benign condition there is uncertain malignant potential with each case requiring individual evaluation to determine the best course of care.

IMT's have been most commonly reported in patients aged 40–55 years old though they have been seen in patients as young as 3 years old and in patients aged up to 89 years old.^8^ Hematuria is the most common presenting symptom, although urinary frequency, dysuria and obstructive symptoms may also occur.

Histologically, IMT's can present in three patterns, often with more than one pattern in a single lesion. The first pattern appears like granulation tissue with nodular fasciitis and spindle cells arranged in a myxoid pattern with eosinophilic cytoplasm. The second recognized pattern is predominantly inflammatory with a higher percentage of plasma cells and lymphocytes and the third is hypocellular with a larger collagenous component.^9^ Generally, these lesions can be described as an atypical spindle cell proliferation in a dense or loose myxoid stroma with or without a background of inflammatory cells. These histological features overlap with malignant spindle cell tumors, aberrant inflammatory processes, leiomyomas and leiomyosarcomas, highlighting the difficulty in their diagnosis for clinicians.^10^

Immunohistochemical markers have also been investigated with a wide range of positive stains including Cytokeratin (AE1/AE3), Smooth Muscle Actin, Vimentin, ALK-1, MIB-1, SV40, alpha-Actin, Calponin, Clusterin and P53.^8^^,^^11^ Frequent positive staining for ALK-1 proteins gained attention and led investigators to analyze the lesions for ALK gene rearrangements with up to 67 % of cases positive for these rearrangements.^12^ This is a significant finding because ALK rearrangement is known to be associated with mesenchymal and lymphoid neoplasms, suggesting that ALK + IMT's represent a true neoplastic process.^3^ Additionally, ALK-1 protein expression has not been reported in leiomyosarcomas and sarcomatoid urothelial carcinomas, making ALK-1 useful in the differentiation of IMT's from other spindle cell lesions.^12^ Overall, there is no significant difference in the progression, malignant potential or rate of recurrence based on ALK-rearrangement status.

Treatment of these lesions has largely consisted of TURBT, partial cystectomy, and rarely radical cystectomy. These tumors generally do not recur and only have rarely been reported to metastasize.^7^ The success of treatment is largely dependent on achieving negative tumor margins with resection. As with many rare disease states, more cases need to be reported to appropriately stratify their level of risk and determine the most appropriate treatment modality.

## 4 Conclusion

Inflammatory Myofibroblastic Tumors are rare neoplasms that are infrequently encountered in the genitourinary tract. Due to their low prevalence, spectrum of lesion types and variable clinical course, they remain a diagnostic and therapeutic challenge to current clinicians. Diagnosis of IMT's can be made through biopsy with histological evaluation. Immunohistochemical markers have been a recent focus of research in an effort to classify the malignant potential of these lesions, but clear markers indicative of clinical course have yet to be identified. Treatment typically consists of local resection which is often curative, but recurrence and metastasis have been reported. Further research is needed to develop definitive treatment guidelines, however our patient in this case was successfully treated with cystoscopic resection.

## CRediT authorship contribution statement

**Samuel Lassiter:** Investigation, Writing – original draft, Writing – review & editing. **Michael L. Smith:** Conceptualization, Data curation, Formal analysis, Writing – review & editing. **James Siegert:** Conceptualization, Data curation, Supervision. **Thai Nguyen:** Conceptualization, Data curation, Supervision.

## Declaration of competing interest

None.
